# The Use of Equimolar Mixtures of Nitrous Oxide and Oxygen in Oral Surgery—A Retrospective Study of Patients in a Swiss University Hospital Setting

**DOI:** 10.3390/jcm12124117

**Published:** 2023-06-18

**Authors:** Alexandre Perez, Steven Gernandt, Paolo Scolozzi

**Affiliations:** 1 Unit of Oral Surgery and Implantology, Division of Oral and Maxillofacial Surgery, Department of Surgery, Faculty of Medicine, Hôpitaux Universitaires de Genève, 1211 Genève, Switzerland; alexandre.perez@hcuge.ch; 2 Division of Oral and Maxillofacial Surgery, Department of Surgery, Faculty of Medicine, Hôpitaux Universitaires de Genève, 1211 Genève, Switzerland; steven.gernandt@hcuge.ch

**Keywords:** nitrous oxide inhaled sedation, oral surgery, dental anxiety

## Abstract

Purpose: The purpose of the study was to evaluate the success of procedural conscious sedation using inhaled equimolar nitrous oxide–oxygen (NOIS—EMONO) in patients undergoing routine dental and oral surgery procedures in a Swiss university hospital setting. Materials and methods: The authors conducted a retrospective cohort study of patients that underwent NOIS-supported procedures between 2018 and 2022 at the oral surgery department of the University Hospital of Geneva (HUG), Switzerland. The primary outcome was the measurement of the procedure’s success and efficacy as defined by the European Society of Anesthesiology. Secondary objectives included the analysis of the types of treatments performed, their indications, patient behavior, and the patient–clinician satisfaction score. Results: 55 patients were included in the study; 85% underwent surgical procedures, and the remaining 15% underwent restorative and preventive procedures. The overall treatment success rate was 98.2% and 97.9% for surgically treated patients. Out of the patients, 62% appeared relaxed, calm, and serene, while 16% expressed pain or fear during the procedure. Infiltrative administration of local anesthesia caused stress in 22% of patients. This portion was significantly lower in sub-cohorts who received local topical anesthetics (0%) or a combination of systemic and local topical analgesics (7%). Patients (75%) and clinicians (91%) were satisfied with the procedure. Conclusion: Inhaled equimolar nitrous oxide–oxygen procedural sedation used during dental procedures and oral surgery results in high treatment success and satisfaction rates. The administration of additional topical anesthetics helps to reduce the anxiety and stress related to infiltrative anesthesia. Further dedicated studies and prospective trials are needed to confirm these findings.

## 1. Introduction

Dental-related anxiety is a common phenomenon reported to affect up to 20% of the adult population [1]. Affected patients may avoid routine examinations or treatment, resulting in undiagnosed dental problems and exacerbating existing oral health issues. This can result in compromised dental function, inadequate oral hygiene, and associated psychosocial impairments or systemic health problems. These factors may profoundly impact a patient’s overall quality of life [2,3].

Various pharmacological and non-pharmacological tools have been developed to address dental anxiety and facilitate the treatment of phobic patients. Nitrous oxide–oxygen inhaled sedation (NOIS) represents one of the most effective and well-established pharmacological approaches to reducing anxiety and pain during dental treatment [3,4,5]. NOIS involves the administration of an equimolar mixture of nitrous oxide and oxygen (EMONO), which has been shown to induce a state of analgesia, anxiolysis, and muscle relaxation by acting on type A γ-aminobutyric acid (GABAA) and N-methyl-D-aspartate (NMDA) glutamate receptors [6]. The use of NOIS has been demonstrated to increase the pain threshold and reduce anxiety in patients undergoing dental procedures [7,8].

Dental anxiety may be regarded as complex and influenced by a multitude of factors—e.g., the type and invasiveness of the procedure, the patient’s past experiences, and the clinical setting in which the treatment is provided [8,9,10,11,12,13,14]. Other factors include: the interaction and management of the patient during treatment, the patient’s medical history, the duration and type of procedure, the patient’s level of cooperation, and the skills of the dental team [8,9,10,11]. Despite a fairly broad literature base describing the efficacy of EMONO-based NOIS-mediated dental treatments, the multifactorial influence affecting treatment outcomes may restrict the overall validity of reported outcomes to alternatives despite similar clinical settings and patient cohorts [8,12].

This retrospective cohort study aimed to describe and evaluate the hitherto unreported success of inhaled equimolar nitrous oxide–oxygen procedural sedation in patients undergoing routine dental with a specific focus on oral surgery procedures over a three-and-a-half-year period in a Swiss university hospital setting.

## 2. Materials and Methods

### 2.1. Study Design

This retrospective study aimed to evaluate the success of a nitrous oxide–oxygen procedural sedation (NOIS) using an inhaled nitrous oxide/oxygen (N_2_O/O_2_) 50–50% equimolar mixture (EMONO) for patients undergoing routine dental and surgical treatments in a Swiss university hospital setting. The study also aimed to analyze the dental procedures by indication and type, the patient’s behavior, and the patient’s and clinician’s satisfaction. Additionally, the study analyzed the frequency and type of additional local and systemic analgesics administered prior to infiltration anesthesia and their potential impact on the patient’s behavior and typical adverse events. The evaluation was restricted to existing standard data records within the University Hospital’s quality systems. No additional measurements, questionnaires, or scores were generated or used for this study. Treatments and reporting adhered to the Helsinki Declaration of ethical principles by the World Medical Association. This study was approved by the Institution’s Ethical Review Board (IRB) (Commission Cantonale d’Ethique de la Recherche sur l’être humain, Geneva, Switzerland, (CCER)) under the approval number 2020-00997.

### 2.2. Study Population

The studied population included all patients who underwent NOIS-supported dental procedures between September 2018 and May 2022 at the oral surgery department of the Geneva University Hospital, Switzerland. The inclusion criteria were patients diagnosed with a condition requiring dental or oral surgery interventions, who were classified as ASA class I or II and were unable to undergo such procedures with routine local anesthesia due to treatment-related anxiety, extremely low pain tolerance, intellectual/cognitive disability, or a young age with uncooperative behavior. All patients or legal guardians gave their informed consent before treatment. Patients with a history of or currently presenting with intracranial hypertension, spontaneous or traumatic pneumothorax, emphysema, abdominal distension due to gas accumulation, intestinal obstruction, sinusitis, ear infection, post-surgical complications of the middle ear, recent sinus-treatments, maxillofacial trauma or fractures, first-trimester pregnancy, non-compliant patients, and those unable to wear masks were excluded from treatment. Patients were instructed to refrain from eating for at least 2 h before the planned procedure.

### 2.3. Treatment Protocol

The treatment protocol followed the standard operating procedures of the University Hospital of Geneva. NOIS administration and treatments were restricted to assistants and clinicians specifically trained in this type of conscious sedation and certified in cardio-respiratory life-support and resuscitation. EMONO was administered via a nasal mask (Accutron™ PIP+™, Accutron, Chicago, IL, USA) at a flow rate of 12 L/min or 6 L/min for patients weighing less than 30 kg. Patients were instructed to operate the mask before treatment, and information on possible adverse events was reinforced. The assistant performed mask administration in disabled patients or children. All patients were continuously monitored for vital signs, including heart rate, oxygen saturation, and any signs or expressions of pain throughout the procedure. Parents and accompanying persons were allowed to be present in the operating theatre. Treatment was started after an induction period of at least 3 min, and active verbal contact with the patient was maintained throughout the procedure.

### 2.4. Data Collection and Analysis

Data collection included demographic data—such as age, gender, and pregnancy status—as well as data related to the evaluation of cognitive abilities, history of NOIS-supported treatments, patient history including relevant systemic conditions, allergies, medical risks, and contraindications related to dental treatments and the administration of EMONO. Recorded treatment-related aspects included the duration of the procedure, the use of self-administered systemic analgesics, the administration of topical anesthetics in addition to infiltration anesthesia, the patient’s attitude before inhalation, the duration of the sedation, and the blood oxygen saturation during the procedure.

Treatment and NOIS administration were evaluated as successful and effective based on the European Society of Anesthesiology’s definition. They were evaluated in conjunction with the patient’s behavior and post-therapeutic evaluation [15].

The degree of sedation was determined using the Ramsay Sedation Scale, as per the European Society of Anesthesiology’s guidelines, by the treating clinical assistant targeting a sedation level 2 corresponding with the patient being cooperative, calm, and oriented, with a tranquil response to stimuli [15].

The patients were further observed for their behavior and mental state during the NOIS-supported treatment. The assistant rated the perceived qualitative tolerance to treatment by assigning one or several of the following attributes: calm and serene, stressed at the moment of local anesthesia administration, worried/panicking, visually expressing signs of pain or fear, a tendency to reject mask, a tendency to reject treatment. Patient and clinician satisfaction was recorded after verbal interrogation 10 min after treatment completion by allowing the choice between one of the following attributes: completely satisfied, partly satisfied, not satisfied, or not able to judge.

Finally, any obligation to interrupt the treatment was recorded, and any patient-reported adverse effects immediately after treatment and 10 min after cessation of the administration of EMONO were noted. Patient and clinician self-reported satisfaction was also recorded 10 min after treatment.

Data analysis and presentation were purely descriptive. Statistical comparisons of potential differences between patient sub-cohorts were not in the scope of the present study, except for exploratory comparisons of sub-cohorts receiving and not receiving additional local anesthetics. These exploratory comparisons used the Fisher’s exact test, assuming outcomes with a *p*-value < 0.05 as statistically significant.

## 3. Results

### 3.1. Cohort Characteristics

During the defined study period of 3.5 years, the records of 55 patients that underwent dental treatment under NOIS utilizing EMONO were analyzed for this study. These patients were between 3 and 60 years old, averaging 23.22 ± 14.4 years. Among the patient cohort, 56% were women, 91% of patients had no cognitive impairment, and of the remaining 9%, 1 patient was autistic, 2 patients were affected by unspecified psychomotor retardation, 1 had Trisomy 21, and 1 was a 3-year-old pediatric patient. In total, 12 pediatric patients under 12 years were included in the study.

Of the total patient cohort, 24% (*n* = 13) had previously undergone NOIS-supported dental or medical treatments, while 70% (*n* = 40) were treated for the first time. Most patients were in good general health (98%, *n* = 54) (ASA class I), with only 1 patient (2%) affected by exercise-induced asthma (ASA class II). None of the patients displayed any treatment-associated risk factors, as shown in (Table 1).

### 3.2. Indications

The success rate, evaluated by the ability to complete and successfully deliver the planned dental treatment, was 98.2%. One patient, who was scheduled for multiple third molar extractions, could not undergo treatment due to severe anxiety, despite the administration of EMONO. The success rate of patients treated surgically was 97.9%.

The absolute numbers and frequencies of treatments, stratified by type and indication, are reported in (Table 2).

Most dental procedures were classified as surgical (*n* = 47, 85%). The most performed surgery involved the extraction of non-erupted third molars. A total of 60 single and multiple teeth were removed. Four patients underwent surgical exposure of impacted teeth (7%), and 4 had oral soft tissue biopsies accounting for 7% of procedures in total. Single and multiple extractions of 21 erupted teeth represented the second most frequent surgical procedure (22%). Other nonsurgical procedures included dental hygiene procedures (5%), impressions (4%), endodontic treatments (4%), and orthodontic treatments (2%), which were overall less frequent (total patients *n* = 8, 15%).

### 3.3. State Prior to Treatment and NOIS Administration

Thirty-six patients (65%) reported feeling anxious prior to treatment and NOIS administration; 31 (56%) patients were calm and relaxed, while eight patients (15%) appeared to be uneasy and tense before treatment (Table 3). It is important to note that patient records could contain more than one attribute per patient.

### 3.4. NOIS Inhalation

The interventions in this study lasted an average of 44.55 ± 18.57 min, ranging from 10 to 90 min. Most of the interventions were almost entirely covered by EMONO inhalation, with an average of (42.54 ± 19.87) minutes and a range of 10 to 100 min. The patients’ oxygen saturation levels were always within a normal range, with an average maximum of 99.23 ± 0.73% and an average minimum of (97.56 ± 1.12)%, and never below 94% (Table 4).

### 3.5. Concomitant Analgesics

The use of locally applied topical anesthetics or systemic self-administered anxiolytics or analgesics, in addition to local infiltration anesthesia, is reported in (Table 5).

Local infiltration anesthesia was performed in all patients except in one case (dental impression). Before local infiltration anesthesia, 12 patients (22%) were given local topical analgesics (Xylocain HCl), and five patients (9%) had taken Morphine derivatives, Lorazepam, or Tramadol. Most patients (65%, *n* = 36) received no additional analgesics. Only 2 patients (4%) received a combination of topical anesthetics and systemic analgesics in addition to EMONO. 

### 3.6. Patient Treatment State

The evaluation of the patient’s physical state and level of cooperation for the total cohort and the sub-cohorts as stratified by the use and administration of additional analgesics is reported in Table 6.

It shows that most patients in the cohorts appeared relaxed, calm and serene (62%, *n* = 34). Stress by local anesthesia was the second most frequently recorded state (22% of total patients), and 16% of patients expressed pain or fear. Panicking or the tendency to reject the mask or treatment were rarely noted (4%, 2% and 5% of patients, respectively). Interestingly, the relative proportion of patients that appeared to be stressed by the local anesthesia was distinctly lower in the sub-cohorts receiving additional local topical anesthetics (0 out of 10 patients (0%)) or a combination of systemic analgesics and local topical anesthetics (1 out of 15 (7%)), compared to the sub-cohort of patients that did not receive any extra analgesics (11 out of 40 patients (28%)). Exploratory comparisons of the ratio of patients appearing stressed during local anesthesia in the sub-cohorts with and without additional local topical anesthetics indicated a borderline significant effect of local topical anesthesia (*p* = 0.09). Likewise, the application of topical analgesics also appeared to influence the frequency of patients with a calm and serene expression in a borderline significant manner when comparing the corresponding sub-cohorts (*p* = 0.07).

### 3.7. Adverse Effects

As reported in (Table 7), paresthesia in the hands and fingers was the most frequently patient-reported side effect (*n* = 22, 40%). Seven patients (13%) further reported a feeling of euphoria, while all other side effects—including crying (5%), vertigo (2%), drowsiness (7%), agitation (7%), and hallucinations (4%)—remained below a threshold of 10%. All side effects were reported to have completely vanished 10 min after ending EMONO inhalation.

### 3.8. Patient and Clinician Satisfaction

Patients (*n* = 41, 75%) and clinicians (*n* = 50, 91%) were overall satisfied with the procedure. None of the patients or clinicians expressed complete dissatisfaction (Table 8). Ten (18%) patients could not give their judgement, and 2 (4%) were only partly satisfied. The clinicians were unsatisfied in one instance (2%); 4% of patients’ and 7% of clinicians’ evaluations were unreported.

## 4. Discussion

This retrospective descriptive study conducted in a Swiss university hospital setting evaluated the success of nitrous oxide–oxygen procedural sedation (NOIS) during dental and surgical procedures using an inhaled nitrous oxide/oxygen (N_2_O/O_2_) 50–50% equimolar mixture (EMONO). The study analyzed various treatment aspects, including treatment-tolerability-related aspects, patient behavior, patient–clinician satisfaction, and the treatment type and indication. The patient cohort consisted of a convenience sample undergoing NOIS-supported therapeutic or preventative dental interventions due to an over-proportional anxiety level and limited anxiety pain tolerance at the oral surgical unit of the HUG. Patient selection was not based on specific criteria, resulting in a cohort considered representative of the corresponding regional patient pool of the university hospital.

This retrospective descriptive study evaluated the success of nitrous oxide–oxygen procedural sedation (NOIS) using an inhaled nitrous oxide/oxygen (N_2_O/O_2_) 50–50% equimolar mixture (EMONO) during dental and surgical procedures of dental treatment anxious patients within a Swiss university hospital setting. Besides treatment completion-related success, this study also examined various treatment delivery-related aspects—including patient tolerability and behavior and patient–clinician satisfaction—considering the corresponding treatment type and indication.

Patient and treatment-related factors, including age, mental status, previous treatment experience, treatment indication, patient management and co-administration of analgesics or anxiolytics, among others, have all been reported to affect treatment outcome and success of NOIS-supported dental treatments [3,8,9,10,11,12]. The patient cohort analyzed within this study consisted of a convenience sample considered representative of the corresponding regional patient pool of the university hospital without applying any filter or selection criteria to the analyzed data set. Consequently, a comprehensive analysis of the observed results, considering the various aspects potentially affecting treatment outcomes, may help interpret the observed outcomes adequately.

### 4.1. Main Results and Clinical Relevance

This study revealed a notable success rate of 98.2%, affirming the effectiveness of nitrous oxide/oxygen inhalation sedation (NOIS) in dental treatments. These results align with previous research studies and are consistent with the outcomes of a recent systematic review conducted by Rossit et al., who reported an overall efficacy rate of 95% (CI: 88.8–98.9%) [5,8,12,16].

Rossi et al. reported higher treatment efficacies in adults compared to pediatric patients, with corresponding efficacy rates of 99.9% and 91.9%, respectively. With this regard, the patient cohort undergoing NOIS-supported dental treatments analyzed herein was relatively young and included both adult and pediatric patients. The median and interquartile ranges were 19 and 13.25 to 28.25 years, respectively. Despite these literature-reported differences in treatment efficacy, our observations did not reveal any indications for different outcomes between adult and pediatric patient groups.

Furthermore, recent clinical studies have provided evidence regarding the efficacy of nitrous oxide/oxygen inhalation sedation (NOIS) in mentally healthy and intellectually disabled patients [10,16]. These findings align well with the observations made in the patient cohort analyzed in this study when differentiating between the corresponding sub-cohorts.

Another important factor potentially affecting treatment outcomes is related to previous experience with NOIS, which was reported to improve patient satisfaction and collaboration during dental procedures compared to NOIS-treatment naive patients [7,17]. Within the patient cohort analyzed in this study, approximately a quarter of the patients reported having previous dental treatment experience with NOIS. This prior experience may have played a role in contributing to the overall high success rates observed in this study.

Lastly, the combination of NOIS and systemic analgesics was reported to potentially influence pain and anxiety whilst heavily depending on specific combinations and dosages [6,18]. To this extent, the herein analyzed patient cohort may be considered limited to allow for a clear interpretation of the results regarding this specific aspect.

### 4.2. Treatment Success in Oral Surgical Procedures

Invasive oral surgical procedures and dental extractions have been ranked as the most anxiety provoking among dental patients [9]. Regarding the level of invasiveness, most dental procedures delivered under NOIS to the herein-analyzed patient cohort could be classified as invasive and surgical (85%). Restorative procedures represented a small proportion of delivered treatments. A relevant reported observation within this study was related to the high success rate of 97.9% in the sub-cohort of surgically treated patients.

Berge et al. recently demonstrated the efficacy of nitrous oxide as part of a large oral surgical patient cohort and have described its application as reliable, efficient, and safe for use as part of oral surgical procedures in adult and pediatric patients [19]. However, the authors reported that sedation was either not accepted in 4.1% and 10.4% of patients, or procedures experienced procedural complications, respectively. Sandhu et al. have recently proven that inhalation of titrated nitrous oxide–oxygen with an N_2_O-concentration of up to 67% could reduce stress and stress-associated biomarkers such as cortisol during lengthy periodontal surgical procedures [20].

The efficacy of EMONO as part of surgical procedures was demonstrated and documented as part of two large multicenter trials comprising relevant surgically treated patient sub-cohorts [8,12]. Collado et al. analyzed the treatment success of NOIS-moderated surgical procedures. However, the authors focused on the effect of the clinicians’ experience level and did not report a specifically differentiated analysis of success rate in invasive oral surgical procedures [12]. Hennequin et al., on the other hand, have recently reported a relatively pronounced failure rate of 10.5% for extractions and oral surgery under local anesthesia and EMONO-mediated NOIS when conducted in a private practice setting. To this extent, the herein reported results report for the first time corresponding results for the success rate of NOIS-mediated EMONO application as part of oral surgical interventions within a university hospital setting.

### 4.3. Potential Effects of Additional Topical Analgesics

Another interesting potential finding of this study was related to the observation that the concomitant use of topical anesthetics helped to alleviate the stress and anxiety related to infiltration anesthesia injection. Anxiety related to anesthetic injection has been reported to represent one of the most important psychologic responses in dentally anxious patients [9,21]. Jacobs et al. have shown that NOIS can significantly reduce injection-related pain during inferior alveolar nerve block anesthesia, indicating that NOIS alone—i.e., without administering additional analgesics—has proven effective in moderating these treatment-related fears [13]. Interestingly, this study showed a distinct difference in the proportion of patients that presented signs of stress associated with the local infiltration anesthesia between patients that received additional topical Xylocaine anesthetic in combination with NOIS, and patients receiving NOIS alone.

Exploratory statistical analysis indicated a borderline significant effect of administering additional topical local anesthesia on the patient’s affect. Further, none of the patients receiving additional topical local anesthesia had previously received NOIS treatment, a situation that could have led to confounding. The observed differences and associated potential of concomitant topical anesthesia, when applied in conjunction with NOIS to reduce injection-related stresses in dentally anxious patients, appear to our knowledge as novel. Given the descriptive nature of this study, the presented results should be regarded as preliminary but potentially meaningful to be substantiated as part of future dedicated studies to investigate this patient-relevant aspect further.

### 4.4. Tolerability and Patient Satisfaction

The results of this study and the total absence of serious adverse events also confirmed the well-documented high safety profile related to the use of EMONO-induced NOIS-supported dental treatment [8]. This study’s most frequently observed side effects included paresthesia, euphoria, and the sensation of inebriation. These side effects represent commonly reported minor side effects related to EMONO-induced NOIS and were completely resolved in all patients within 10 min after ending the administration [12,18]. Whilst adverse event-related outcomes of this study appeared comparable to previous reports, methodological disparities and patient cohort characteristics have been described to affect adverse event outcomes, potentially limiting the comparability between studies, specifically for the registration of minor adverse events [22].

Finally, patient and clinician satisfaction with the treatments was overall high. In this regard, it must be considered that satisfaction and anxiety-related outcomes for the type of procedures and patient pools may not be associated with NOIS alone. However, other factors, such as the clinical setting and patient-experience-specific psychophysiological component beyond NOIS application, as well as the application of anxiety and pain management techniques provided by the dentist or assistant, need to be taken into account for the adequate interpretation of such outcomes [3,11,12,14].

### 4.5. Limitations

The herein reported results require consideration of the limitations related to the descriptive study setup and limited patient number, potentially allowing overall confirmatory and limited analytical statements. Patient numbers were limited by the relatively low estimated percentage of 0.3% of dental patients in our centre undergoing this type of treatment.

## 5. Conclusions

This retrospective descriptive study was the first documented that conscious sedation with an inhaled equimolar mixture of nitrous oxide and oxygen is a highly effective, successful and satisfactory treatment option for routine dental treatment-anxious patients in a Swiss hospital university setting. The success rate was notable, particularly in surgical and invasive procedures, highlighting the benefits of this sedation technique in oral surgical procedures. Additional topical anesthetics may help alleviate the anxiety and stress related to infiltration anesthesia injections, which may be substantiated in future trials.

## Figures and Tables

**Table 1:** Patient-related descriptive characteristics. Age is reported as an average and standard deviation. All other parameters are reported as a percentage relative to the patient cohort.

Characteristic	Parameter	*n*	Mean ± SD/%
Age [years]	Age at Intervention	55	23.22 ± 14.4
Gender	Gender (%Men)	24	44%
	Gender (%Women)	31	56%
Cognitive capabilities	Normal cognitive capabilities	50	91%
	Altered cognitive capabilities	5	9%
Prior treatment exposure	None/first treatment	40	73%
	Prior exposure	13	24%
	Not assessed	2	4%
ASA physical status classification	ASA class I	54	98%
	ASA class II	1	2%
	ASA class ≥ III	0	0%
Treatment-associated risk factors	Present	0	0%
	Not present	55	100%

**Table 2:** Provided treatments per procedure type and indication. The reported percentage is based on the total patient cohort. ^†^ Number in brackets refers to the total number of extracted teeth.

Type	Indication	*n*	%
Surgical including flaps		35	64%
	Oral soft tissue biopsy	4	7%
	Surgical exposure of impacted tooth	4	7%
	Third Molar extraction, non-erupted, multiple ^†^	18 (60)	33%
	Third Molar extraction, non-erupted, single	1	2%
	Tooth extraction, non-erupted, single	8	15%
Surgical without flaps		12	22%
	Dental extraction, multiple ^†^	10 (21)	18%
	Dental extraction, single	2	4%
Other procedures		8	15%
	Dental hygiene procedure	3	5%
	Dental Impression taking	2	4%
	Endodontic treatment—multiple teeth	1	2%
	Endodontic treatment—single tooth	1	2%
	Orthodontic treatment	1	2%

**Table 3:** Clinician or assistant-judged patient state prior to treatment and NOIS administration. The reported percentage refers to the relative portion of patients in the total cohort. Note that patient records could contain more than one attribute per patient.

Patient State	*n*	%
Calm, relaxed	31	56%
Anxious	36	65%
Uneasy, tense	8	15%
Not reported	3	5%

**Table 4:** NOIS administration-related characteristics, comprising the EMONO inhalation time, the total intervention duration, and the minimum and maximum oxygen blood saturation values during treatment. Values are reported as averages, standard deviations, medians, interquartile, and total ranges. Abbreviations: SD—standard deviation; IQR—interquartile ranges.

Parameter	*n*	Mean ± SD	Median (IQR)	Range
Duration of the intervention [min]	55	44.55 ± 18.57	45 (30–60)	10–90
Inhalation time [min]	54	42.54 ± 19.87	45 (25–60)	10–100
Oxygen Saturation [max] [%]	53	99.23 ± 0.73	99 (99–100)	97–100
Oxygen Saturation [min] [%]	53	97.56 ± 1.12	98 (97–98)	94–99

**Table 5:** Administered topical anesthetic or self-administered systemic analgesics/anxiolytics in addition to local infiltration anesthesia, organized by type and active substance. Reported percentages refer to the relative portion of patients in the total cohort. Note that patient records could contain more than one attribute per patient.

Type	Active Substance and Dosage Form	*n*	%
Topical anesthetic	None	43	78%
	Xylocaine HCl Gel	9	16%
	Xylocaine HCl Spray	3	5%
Systemic analgesics/anxiolytics	None	50	91%
	Morphine	1	2%
	Lorazepam	3	5%
	Tramadol	1	2%
Combined topical and systemic	Combined	2	4%
	Only topical	12	22%
	Only systemic	5	9%
	No systemic or topical	36	65%

**Table 6:** Clinician or assistant-judged patient state per total cohort and sub-cohorts receiving no additional analgesics, topical anesthetics, systemic analgesics, or a combination. Reported percentages refer to the relative portion of patients in the cohort or corresponding sub-cohort. Note that patient records could contain more than one attribute.

Patient Condition	Total Cohort (*n* = 55)		Sub-Cohort Topical Anesthetic (*n* = 10)		Sub-Cohort Analgesics (Local and/or Systemic) (*n* = 15)		Sub-Cohort without Analgesics (*n* = 40)	
Category	*n*	%	*n*	%	*n*	%	*n*	%
Calm and serene	34	62%	9	90%	12	80%	22	55%
Stressed by local anesthesia	12	22%	0	0%	1	7%	11	28%
Worried—panicked	2	4%	0	0%	0	0%	2	5%
Visual expression of pain or fear	9	16%	2	20%	4	27%	5	13%
Tendency to reject mask	1	2%	0	0%	0	0%	1	3%
Tendency to reject treatment	3	5%	0	0%	0	0%	3	8%

**Table 7:** Number and relative percentages of observed side effects. Reported individual side effects refer to the total patient cohort. Please note that a patient could have displayed more than one side effect.

Side Effect	*n*	%
None	13	24%
Not reported	4	7%
Paraesthesia	22	40%
Crying	3	5%
Vertigo	1	2%
Drowsiness, sleepiness	4	7%
Agitation	4	7%
Sensation of inebriation	5	9%
Hallucination	2	4%
Euphoria	7	13%

**Table 8:** Patient and clinician-reported satisfaction after treatment. Reported percentages refer to the total patient cohort.

Category	Patient Satisfaction		Clinician Satisfaction	
	*n*	%	*n*	%
Not reported	2	4%	4	7%
Completely satisfied	41	75%	50	91%
Partly satisfied	2	4%	1	2%
Not satisfied	0	0%	0	0%
Not able to judge	10	18%	0	0%

## Data Availability

The authors declare that the data supporting the findings of this study are available within the paper.
